# *Citrus limonL*.-Derived Nanovesicles Show an Inhibitory Effect on Cell Growth in p53-Inactivated Colorectal Cancer Cells via the Macropinocytosis Pathway

**DOI:** 10.3390/biomedicines10061352

**Published:** 2022-06-08

**Authors:** Hideki Takakura, Toshimasa Nakao, Takumi Narita, Mano Horinaka, Yukako Nakao-Ise, Tetsushi Yamamoto, Yosuke Iizumi, Motoki Watanabe, Yoshihiro Sowa, Keisuke Oda, Nobuhiro Mori, Toshiyuki Sakai, Michihiro Mutoh

**Affiliations:** 1 Department of Molecular-Targeting Prevention, Kyoto Prefectural University of Medicine, Kyoto 602-8566, Japan; takakura@hirokoku-u.ac.jp (H.T.); toshi876@koto.kpu-m.ac.jp (T.N.); narita@koto.kpu-m.ac.jp (T.N.); m-hori@koto.kpu-m.ac.jp (M.H.); nakaoy@koto.kpu-m.ac.jp (Y.N.-I.); yiizumi@koto.kpu-m.ac.jp (Y.I.); mtkw@koto.kpu-m.ac.jp (M.W.); ysowa@koto.kpu-m.ac.jp (Y.S.); tsakai@koto.kpu-m.ac.jp (T.S.); 2 Department of Drug Discovery Medicine, Kyoto Prefectural University of Medicine, Kyoto 602-8566, Japan; 3 Laboratory of Biopharmaceutics and Pharmacokinetics, Faculty of Pharmaceutical Sciences, Hiroshima International University, Hiroshima 737-0112, Japan; oda@hirokoku-u.ac.jp (K.O.); n-mori@hirokoku-u.ac.jp (N.M.); 4 Pathological and Biomolecule Analyses Laboratory, Faculty of Pharmacy, Kindai University, Osaka 577-8502, Japan; yamatetsu@phar.kindai.ac.jp

**Keywords:** colorectal cancer, p53, nanovesicles, edible plant, macropinocytosis, colorectal cancer prevention

## Abstract

Edible plant-derived nanovesicles have been explored as effective materials for preventing colorectal cancer (CRC) incidence, dependent on gene status, as a K-Ras-activating mutation via the macropinocytosis pathway. Approximately 70% of CRC harbors the p53 mutation, which is strongly associated with a poor prognosis for CRC. However, it has not been revealed whether p53 inactivation activates the macropinocytosis pathway or not. In this study, we investigated parental cells, wild-type or null for p53 treated with *Citrus limon L.*-derived nanovesicles, as potential materials for CRC prevention. Using ultracentrifugation, we obtained *C. limon L*.-derived nanovesicles, the diameters of which were approximately 100 nm, similar to that of the exosomes derived from mammalian cells. *C. limon L*.-derived nanovesicles showed inhibitory effects on cell growth in not p53-wild, but also in p53-inactivated CRC cells. Furthermore, we revealed that the macropinocytosis pathway is activated by p53 inactivation and *C. limon L.*-derived nanovesicles were up taken via the macropinocytosis pathway. Notably, although *C. limon L*.-derived nanovesicles contained citrate, the inhibitory effects of citrate were not dependent on the p53 status. We thus provide a novel mechanism for the growth inhibition of *C. limon L*.-derived nanovesicles via macropinocytosis and expect to develop a functional food product containing them for preventing p53-inactivation CRC incidence.

## 1. Introduction

Colorectal cancer (CRC) is one of the most common cancers in the world [1]. From a genomic perspective, the p53 mutation is frequently observed in CRC. p53 is a tumor suppresser gene, but its function is inactivated in many cancers through mutation or loss [2]. In the case of CRC, p53 mutants were observed in about 70% of cancers, and CRC with mutant p53 is strongly associated with a poor prognosis [3]. Therefore, practical methods against p53-inactivated CRC are needed for the prevention of CRC incidence.

From an environmental point of view, it has been reported that dietary habits influence the risk of CRC incidence [4]. Polyphenols and carotenoids, which are components of edible plants, are expected to be effective at preventing CRC [5,6]. Edible plants are important resources of nutrition, and recently some have been shown to have potential as functional foods with various bioactivities [7,8,9]. Exosomes derived from cells are reported to be stable carriers that elicit bioactive signals in mammalian intestinal fluid. Among edible plant extracts, several researchers have found the existence of exosome-like nanovesicles [10]. It has been reported that the oral administration of edible plants involves the uptake of exosome-like nanovesicles by intestinal macrophages and stem cells in mice [11,12]. Edible plant-derived nanovesicles are expected to be used as a stable bioactive cargo for preventing CRC.

Macropinocytosis is an endocytosis system that uptakes exosomes. Macropinocytosis is activated via oncogene activation [13]. For instance, it has been shown that K-Ras activating mutations induce exosome uptake via activating macropinocytosis in several cancer cell lines [14,15]. About 40% of CRC cases are characterized by a mutation in the KRAS gene [16]. However, there are no molecular targeted drugs for KRAS-mutant CRC in FDA approved drugs [17,18]. Therefore, macropinocytosis should be targeted as a novel drug delivery system, especially for cancer cells that have K-Ras-activating mutations. However, it has not been revealed whether tumor suppressor genes, such as for p53 inactivation, induce macropinocytosis activation in cancer cells.

Nanovesicles/nanoparticles are used as an orally administrable drug delivery system to the tumor sites of colorectal cancer. Nanovesicles have been reported to improve selectivity and bioavailability. Doxorubicin-loaded nanovesicles reduced CRC tumor growth without the adverse effects observed using equipotent free drugs in vitro [19]. Polyphenols and carotenoids are known as poor solubility natural products in the intestinal field. Curcumin is a typical polyphenol that has an effective anti-cancer component. Curcumin nanoparticles can be more bioavailable than normal curcumin [20]. Currently, clinical trials regarding the administration of a curcumin conjugate grapefruit-derived nanovesicle for patients with CRC are underway (https://clinicaltrials.gov, NCT 01294072; accessed on 16 May 2022). Thus, clinical applications of, for example, nanovesicles/nanoparticles, will afford various benefits for CRC treatment.

In this study, we focused on *Citrus limon L.*-derived nanovesicles and evaluated whether they could be a novel material preventing against p53-inactivated CRC incidence. This first report showed that edible plant-derived nanovesicles with a cell growth inhibitory effect on CRC cells depend on p53 activation status via the macropinocytosis pathway, and that p53 inactivation induced macropinocytosis activity.

## 2. Materials and Methods

### 2.1. Preparation Nanovesicles

*C. limonL*. was purchased from an organic farmer in Ehime, Japan. *C. limonL.* was gently squeezed manually. The juice was centrifuged at 2000× *g* for 40 min, and the supernatant was centrifuged at 10,000× *g* for 60 min. The supernatant was filtered through a 0.22 μm pore filter and centrifuged at 100,000× *g* for 90 min in a fixed angle rotor Type 45 Ti (Beckman Coulter Inc., Brea, CA, USA). The pellet was suspended in PBS (-) and was transferred to a 30% sucrose/D_2_O cushion. After centrifugation at 100,000× *g* for 180 min in a swinging-bucket rotor SW 32.1 Ti (Beckman Coulter Inc., Brea, CA, USA), the nanovesicle-containing fraction was resuspended in PBS (-) and was centrifuged at 100,000× *g* for 90 min two times. The pellet was collected and resuspended in PBS (-) for the subsequent experiments.

### 2.2. Transmission Electron Microscopy

Nanovesicles were fixed in 2.5% glutaraldehyde, 2% paraformaldehyde, and 1% osmium tetroxide, and then stained with 2% lead citrate. The preparation was examined using a transmission electron microscope (TEM) JEM-1220 (JEOL Ltd., Tokyo, Japan) at 100 kV.

### 2.3. Nanoparticle Tracking Analysis

**Figure 1:**
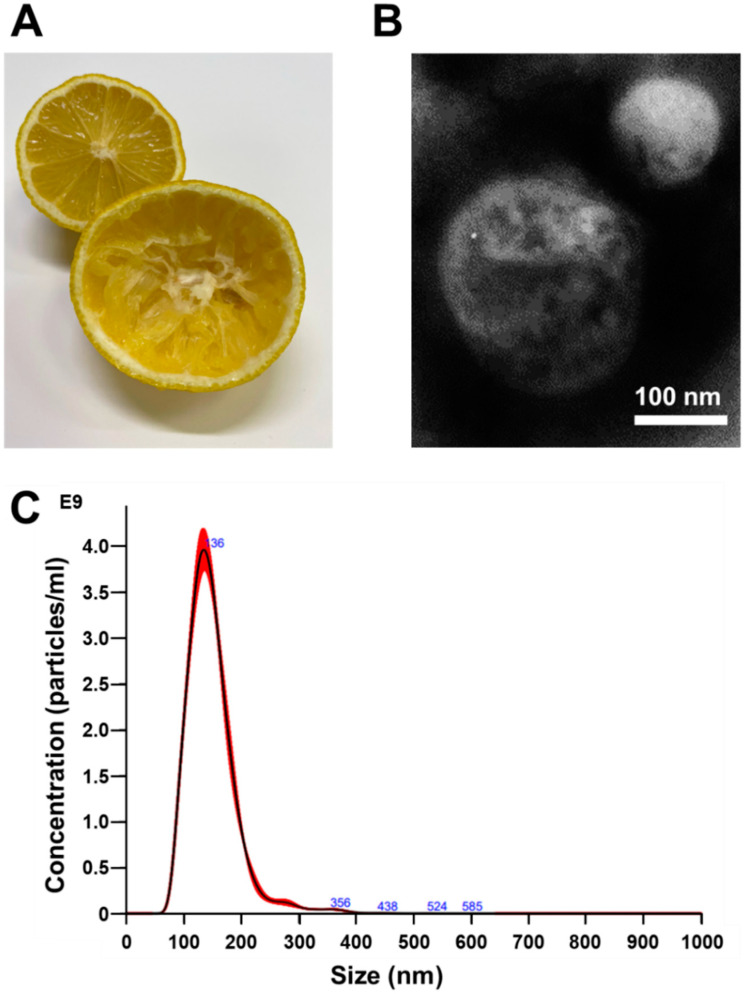
Analysis of the diameter and concentration for *Citrus limon L.*-derived nanovesicles using TEM and NTA. (**A**) Photograph of *C. limon* *L.* before obtaining juice (lemon in the back of the photo) and after obtaining juice by squeezing (lemon in front of the photo). (**B**) Representative photograph of nanovesicles obtained at 50,000× magnification using transmission electron microscopy. Scale bar, 100 nm. (**C**) Size distribution of nanovesicles. Size distributions were determined by the nanoparticle tracking analysis.

Nanoparticle tracking analysis (NTA) was performed using a NanoSight NS300 system (Malvern Technologies, Malvern, UK) configured with a 488 nm laser and a high-sensitivity scientific complementary metal-oxide semiconductor camera. NTA was used to confirm the size distribution and concentration of the nanovesicles. The samples were diluted 100-fold with PBS (-) and were measured using NTA. NTA was performed five times to obtain the average value and standard error (Figure 1C). The measured value was multiplied by the dilution factor to calculate the concentration of nanoparticles in the sample (Table 1).

### 2.4. Resistive Pulse Sensing

Tunable resistive pulse sensing (TRPS) was performed using a qNano (Izon, Cambridge, MA, USA) according to the manufacturer’s instructions. Samples were diluted 50-fold with PBS (-) and were measured using TRPS. The measured value was multiplied by the dilution factor to calculate the concentration of nanoparticles in the sample.

### 2.5. Cell Culture

Human CRC cell lines, HCT-15 and SW480, were purchased as a cell line of NCI-60 from the NCI Developmental Therapeutics Program. HCT116 wild-type (WT) and HCT116 p53 null-type ([-/-]) cells were kindly provided by Dr. B. Vogelstein [2]. These cells were cultured in Roswell Park Memorial Institute 1640 medium with 10% exosome-depleted fetal bovine serum (Thermo Fisher Scientific, Waltham, MA, USA). All of the cells were cultured at 37 °C in an atmosphere containing 5% CO_2_.

### 2.6. Cell Growth Assay

HCT116 WT, HCT116 p53 (-/-), HCT-15, and SW480 cells were plated at a density of 2 × 10^3^ cells per well in 96-well plates. After pre-incubation for 24 h, the cells were treated with *C. limon* *L*.-derived nanovesicles or citrate (Nacalai Tesque, Kyoto, Japan) for 24, 48, or 72 h. After treatment, the cells were incubated with a WST-8 reagent (Cell Counting Kit-8; Dojindo Laboratories, Kumamoto, Japan) for 4 h at 37 °C, and the optical density of the culture solution was measured at 450 nm using an ELISA plate reader.

### 2.7. Labeling Nanovesicles and Uptake for Cells

Nanovesicles were labeled with an ExoSparkler Exosome Membrane Labeling Kit (Dojindo) in accordance with the product information. The cells were plated at a density of 2 × 10^4^ cells per well in six-well plates. After pre-incubation for 24 h, the cells were treated with 0.5 mg/mL 70 kDa FITC-dextran (Sigma-Aldrich, St. Louis, MO, USA) or 5 μg/mL labeled nanovesicles contenting medium for 6 or 24 h. For the fluorescence microscopy (BZ-X800; KEYENCE Co., Osaka, Japan), the nuclei were stained with Hoechst 33342 (Dojindo). For the flow cytometry, the cells were harvested using trypsinization. Dead cells were stained with a fixable viability stain (FVS) (Becton, Dickinson and Company, Franklin Lakes, NJ, USA) for 30 min. Flow cytometric data were acquired using a CantoII flow cytometer (Becton, Dickinson and Company) and were analyzed using FlowJo version 10 software (Tree Star, San Carlos, CA, USA).

### 2.8. Citrate Assay

The citrate concentration was measured using a Citrate assay kit (Sigma-Aldrich) in accordance with the product information. In brief, the enzymes in a *C. limon L.*-derived nanovesicle suspension were excluded using a 10 kDa MWCO spin filter. After the reaction, the citrate concentration was measured at 570 nm using an ELISA plate reader. Reactions of oxaloacetate or pyruvate were removed as a background in the assay.

### 2.9. Intracellular pH Measurement

The cells were plated at a density of 2 × 10^3^ cells per well in 96-well plates. After incubation for 24 h, the cells were treated with *C. limon L.*-derived nanovesicles for 6 h. Then, the cells were washed with PBS (-) and incubated with 3 μM BCECF-AM (Dojindo) for 30 min at 37 °C. BCECF-AM fluorescence was measured using excitation at 440 nm and 490 nm, and emission at 535 nm using an ELISA plate reader. The intracellular pH was calculated using the 490/440 nm fluorescence ratio and standard solutions with pH values.

### 2.10. Statistical Analysis

All data are presented as mean ± standard error measurement. Analysis was performed with GraphPad Prism version 7.0.4 (GraphPad Software, Inc., San Diego, CA, USA). Differences among groups were evaluated using one-way analysis of variance, followed by Dunnett’s multiple comparisons test, Sidak’s multiple comparisons test, or an unpaired *t* test. *p* < 0.05 was considered to indicate a statistically significant difference.

## 3. Results

### 3.1. Isolation and Identification of Nanovesicles from Citrus limon L.

To obtain *C. limon* *L.* juice, we cut and squeezed the fruits gently (Figure 1A). Nanovesicles were isolated from *C. limon* *L*. juice using an ultracentrifugation method. The images of the nanovesicles obtained using a transmission electron microscope (TEM) showed that the obtained fraction contained nanovesicles with a diameter of approximately 100 nm (Figure 1B). Moreover, nanoparticle tracking analysis (NTA) revealed the size distribution and concentration of the nanovesicles (Table 1 and Figure 1C). Tunable resistive pulse sensing (TRPS) was performed to confirm the nanovesicles’ particle diameter and concentration. Figure S1 and Table S1 show that the TRPS results were similar to the NTA result. Based on these findings, we confirmed the acquisition of nanovesicles derived from *C. limon* *L.* and that the diameters of these nanovesicles were similar to those of the exosomes derived from the mammalian cells.

**Table 1:** *Citrus limon L*.-derived nanovesicle concentration and components.

Particle Concentration	Protein Concentration	Citrate Concentration
3.38 × 10^11^ particles/mL	625 μg/mL	0.111 nM

3.38 × 10^11^: the total number of digits is 12, and the first three digits are “338”.

### 3.2. Citrus limon L.-Derived Nanovesicles Cause p53-Dependent Cell Growth Inhibition

**Figure 2:**
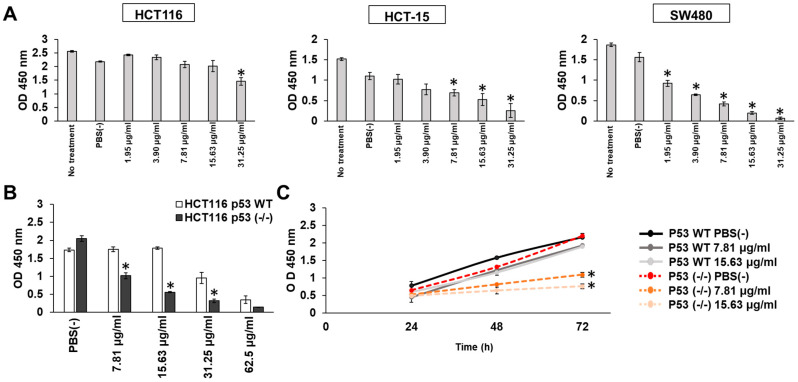
p53 status affects the inhibition of cell growth through *Citrus limon L.*-derived nanovesicle treatment. (**A**) HCT116 WT, HCT-15, and SW480 cells were treated with the indicated concentrations of *C. limon* *L.*-derived nanovesicles for 72 h. (**B**,**C**) HCT116 WT or HCT116 p53 (-/-) cells were treated with the indicated concentrations of *C. limon* *L.*-derived nanovesicles for 24, 48, and 72 h. Cell growth was measured using a Cell Counting Kit-8 assay. Data are means ± standard error measurement; *n* = 3; * *p* < 0.05, relative to HCT116 WT. *p* values were calculated using Dunnett’s multiple comparisons test and Sidak’s multiple comparisons test.

Next, we evaluated the cell growth inhibitory effects of *C. limon* *L.*-derived nanovesicles on human CRC cell lines with K-Ras-activating mutations. HCT116, HCT-15, and SW480 cells have K-Ras-activating mutations [21]. Among the three human CRC cell lines analyzed, HCT116, HCT-15, and SW480 cells, only the HCT-15 and SW480 cells showed an inhibition of cell growth in a dose-dependent manner through treatment with *C. limon L.*-derived nanovesicles for 72 h (Figure 2A). Of note, high dose (31.25 μg/mL) treatment inhibited the cell growth of HCT116 cells. We then focused on the difference in p53 gene status in the tested cell lines; HCT116 cells were p53 WT, whereas both HCT-15 and SW480 cells had mutant p53. To confirm the effects of p53 gene status, we obtained HCT116 p53 (-/-) cells and HCT116 WT cells, and compared the cell growth inhibition induced by *C. limon* *L.*-derived nanovesicles. As expected, *C. limon* *L.*-derived nanovesicles inhibited stronger cell growth in HCT116 p53 (-/-) cells than in HCT116 WT cells in a dose- and time-dependent manner (Figure 2B,C). These results suggest that the inhibitory effects of *C. limon* *L.*-derived nanovesicles on cell growth were dependent on p53 status.

### 3.3. p53 Deficiency Enhance the Uptake of Citrus limon L.-Derived Nanovesicles via Macropinocytosis in HCT116 Cells

**Figure 3:**
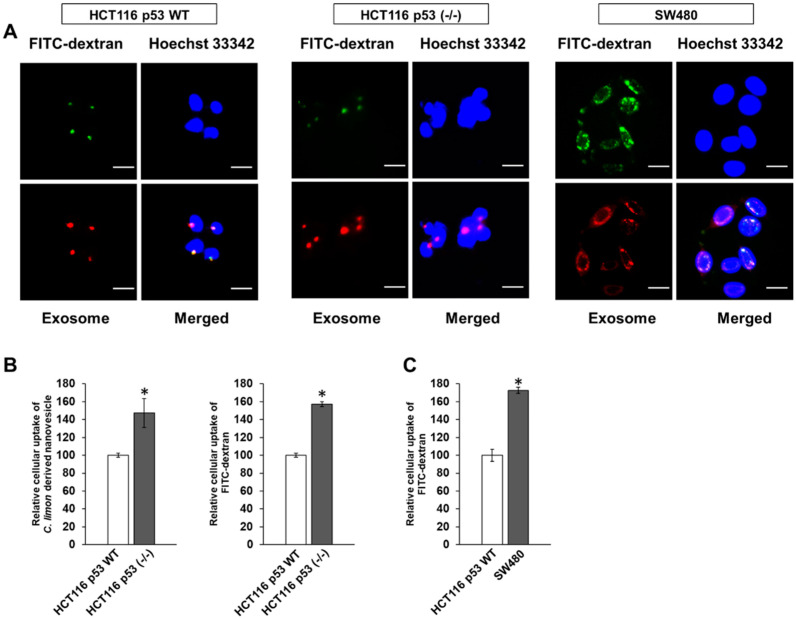
Uptake of *Citrus limon L*.-derived nanovesicles via macropinocytosis in colorectal cancer cells. HCT116 WT, HCT116 p53 (-/-), and SW480 cells were treated with labeled nanovesicles and/or FITC-dextran (70 kDa, macropinocytosis marker) at 37 °C. (**A**) After 24 h, the nuclei were stained with Hoechst 33342. These images were obtained using a fluorescence microscope. Scale bar, 20 μm. (**B**,**C**) After 6 h, relative cellular uptake analysis of labeled nanovesicles or FITC-dextran on these cells through flow cytometry. Data are means ± standard error measurement; *n* = 3; * *p* < 0.05, relative to HCT116 WT. *p* values are calculated using an unpaired *t* test.

Next, we investigated in detail the cellular uptake of *C. limon L*.-derived nanovesicles in CRC cells. FITC-dextran (70 kDa) uptake is a well-known method for evaluating macropinocytosis [14]. As a result, labeled nanovesicles and FITC-dextran were colocalized in the cytoplasm of these cells, especially in the perinuclear region (Figure 3A). Figure 3B shows a strong uptake of labeled nanovesicles and FITC-dextran by HCT116 p53 (-/-) cells compared with HCT116 WT cells. Moreover, the uptake of FITC-dextran was clearly higher in the SW480 cells, a p53 mutant cell line, than the HCT116 WT cells, a p53 WT cell line (Figure 3C). These results suggest that the cellular uptake of *C. limon L*.-derived nanovesicles occurred through the macropinocytosis pathway, whose mechanism is dependent on p53 status.

### 3.4. Citrate Shows p53-Independent Inhibitory Effects on HCT116 Cells

**Figure 4:**
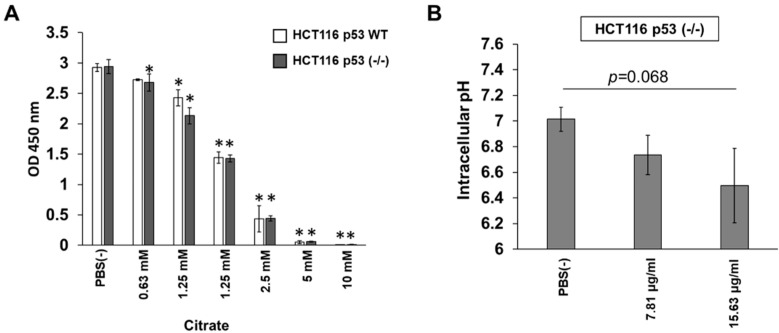
Citrate shows p53-independent inhibitory effects on HCT116 cells. (**A**) HCT116 WT or HCT116 p53 (-/-) cells were treated with the indicated concentrations of citrate for 72 h. Cell growth was measured using a Cell Counting Kit-8 assay. (**B**) HCT116 p53 (-/-) cells were treated with the indicated concentrations of *Citrus limon L.*-derived nanovesicles for 6 h. Intracellular pH was measured using a BCECF-AM assay. Data are means ± standard error measurement; *n* = 3; * *p* < 0.05, relative to the PBS (-) control. *p* values were calculated using Dunnett’s multiple comparisons test and Sidak’s multiple comparisons test.

The characteristic sourness of *C. limon L.* is due to citric acid; therefore, we first examined the growth inhibitory effect of citrate on HCT116 WT and HCT116 p53 (-/-) cells. As expected, citrate showed similar inhibitory effects in both cell lines (Figure 4A). HCT116 p53 (-/-) cells can uptake *C. limon L*.-derived nanovesicles, and we next measured the citrate concentration in *C. limon L*.-derived nanovesicles. As a result, 625 μg/mL of *C. limon L*.-derived nanovesicles contained 0.111 nM citrate (Table 1). Moreover, treatment with *C. limon L*.-derived nanovesicles tended to decrease the intracellular pH in HCT116 p53 (-/-) cells (Figure 4B).

## 4. Discussion

In the present study, *C. limon L.*-derived nanovesicles were shown to possess cell growth inhibitory effects, mainly in p53-inactivated CRC cell lines, through the macropinocytosis pathway.

We first examined the size of *C. limon L.*-derived nanovesicles, because it has been reported that the mechanism of nanovesicle uptake depends on their size [22]. Moreover, if such nanovesicles are to be clinically applied in the future, it would be necessary to confirm that the size of the nanovesicles derived from *C. limon* *L.* was almost the same as those tested in this study. Several reports have confirmed that the diameter of mammalian-derived exosomes is about 100 nm [13,14]. Thus, the diameter of these *C. limon L.* nanovesicles was similar to those of exosomes derived from mammalian cells (Figure 1). Therefore, the uptake pathway of *C. limon L.*-derived nanovesicles into the CRC cell lines was expected to be similar to the uptake pathway of mammalian-derived exosomes.

In our experiment, *C. limon L*.-derived nanovesicles showed growth inhibitory effects in three CRC cell lines that had K-Ras-activating mutations (Figure 2A). As expected, high-dose treatment induced cell growth inhibition, but the dose-dependent inhibitory effects of *C. limon L*.-derived nanovesicles were notably only observed in p53 mutant cells and in p53 (-/-) cells (Figure 2B). Therefore, we used HCT116 WT or HCT116 p53 (-/-) cells to confirm the results and obtained stronger cell growth inhibition in HCT116 p53 (-/-) cells than in HCT116 WT cells (Figure 2C). These results suggest that cells with WT p53 were resistant to the inhibition effects of *C. limon L*.-derived nanovesicles. Furthermore, we speculated that edible plant-derived nanovesicles such as those from *C. limon L*. may be effective at preventing p53-inactivated CRC. p53 mutants have been observed in about 70% of patients with CRC, and CRC with mutant p53 is strongly associated with poor prognosis [3]. Therefore, our findings with p53-inactivated CRC may provide a method for the prevention of CRC treatment.

Next, we examined nanovesicle uptake. Generally, macropinocytosis is proven by FITC-dextran (70 kDa) uptake [14]. We found that FITC-dextran (70 kDa) and labeled nanovesicles were colocalized (Figure 3A), indicating that *C. limon L.*-derived nanovesicles were taken up into the cells via macropinocytosis. Then, we determined why the inhibitory effect of *C. limon L.*-derived nanovesicles depends on p53 status. Our results indicated that the macropinocytosis activity was higher in p53-inactivated cell lines than a p53 WT cell line (Figure 3B,C). Because these results were consistent with the results of the cell growth inhibitory effect, it was considered that the cell growth inhibitory effect was controlled by the amount of *C. limon L.*-derived nanovesicles taken up.

Currently, components of plant derived nanovesicles are collecting a lot of attention as a stable bioactive cargo [23,24]. However, it has not been clarified whether plant derived nanovesicles have a selective inhibitory effect on cell growth for p53-inactivated CRC cell lines. In this article, we would like to focus on and rapidly report that *C. limon L.*-derived nanovesicles possessed cell growth inhibitory effects, mainly in p53-inactivated CRC cell lines, through the macropinocytosis pathway. So, we have not gone into detail regarding the components that inhibit cell growth, but instead only comment on the effects of citrate on cell growth. Citrus fruits are known to be citrate rich, and citrate itself induced cell growth inhibition, but does not have selective inhibitory effect depending on p53 WT or (-/-) (Figure 4A). Recently, *Baldini, N.* et al., reported that *C. limon L.*-derived nanovesicles contain citrate [25]. We checked whether *C. limon L.*-derived nanovesicles contained citrate. However, the amount of citrate (Table 1) was not sufficient to suppress cell growth, as can be seen from the data in Figure 4A. We further examined the intracellular pH in HCT116 p53 (-/-) cells. Notably, HCT116 p53 (-/-) cells with *C. limon L.*-derived nanovesicles tended to show a decrease in intracellular pH from 7 to 6.5 on average (Figure 4B). Small pH changes may affect the homeostasis of cells and have an influence on cell growth. Further examination is needed to reveal the mechanism regarding why the components of *C. limon L.*-derived nanovesicles induce cell growth inhibition. We were unable to observe the accumulation of *C. limon L.*-derived nanovesicles during our short experimental time, but this will be a future issue to confirm.

Edible plant-derived nanovesicles can overcome several problems for cancer treatment and prevention. Macropinocytosis is also known as an amino acid supplement pathway rather than a nanoparticle uptake pathway in cancer cell that have K-Ras-activating mutations [15,26]. High concentrations of amino acids contribute to the overgrowth of cancer cells that have K-Ras-activating mutations [15,26]. Macropinocytosis that confer drug resistance, such as 5-fluorouracil by consuming necrotic cell, have been reported [27]. Edible plant-derived nanovesicles can find novel clinical applications for overcoming high malignancy and drug resistance cancer treatment and prevention.

## 5. Conclusions

In conclusion, the results of the present study indicate that p53-inactivation activated the macropinocytosis activity and *C. limon* *L.*-derived nanovesicles had a cell growth inhibitory effect via the macropinocytosis pathway. Edible plant-derived nanovesicle intake can be incorporated safety in eating habits. These results suggest that *C. limon L.*-derived nanovesicles may provide a means to discover the methods for preventing p53-inactivated CRC incidence.

## Data Availability

Not applicable.

## Supplementary Materials

The following supporting information can be downloaded at: https://www.mdpi.com/article/10.3390/biomedicines10061352/s1, Figure S1: Analysis of the diameter and concentration for *Citrus limon L*.-derived nanovesicles by tunable resistive pulse sensing.; Table S1: *Citrus limon L*.-derived nanovesicle concentration by tunable resistive pulse sensing.
