# Supplementary material for: Citrus limonL.-Derived Nanovesicles Show an Inhibitory Effect on Cell Growth in p53-Inactivated Colorectal Cancer Cells via the Macropinocytosis Pathway
Source: Biomedicines. 2022 Jun 8;10(6):1352. doi: 10.3390/biomedicines10061352 (PMC9219868; doi:10.3390/biomedicines10061352)
Supplement: Supplementary file 1 [file biomedicines-10-01352-s001.zip › biomedicines-1754371-Supplementary.pdf]

biomedicines

MDPI

Article

Supplementary Materials: *Citrus limon L.*-derived nanovesicles show an inhibitory effect on cell growth in p53-inactivated colorectal cancer cells via the macropinocytosis pathway

Hideki Takakura [1,2,3], Toshimasa Nakao [1], Takumi Narita [1] Mano Horinaka [1,2], Yukako Nakao-Ise [1], Tetsushi Yamamoto [4], Yosuke Iizumi [1], Motoki Watanabe [1], Yoshihiro Sowa [1], Keisuke Oda [3], Nobuhiro Mori [3], Toshiyuki Sakai [1,2] and Michihiro Mutoh [1*]

[1] Department of Molecular-Targeting Prevention, Kyoto Prefectural University of Medicine, Kyoto 602-8566, Japan; toshi876@koto.kpu-m.ac.jp (T.N.); narita@koto.kpu-m.ac.jp (T.N.); nakaoy@koto.kpu-m.ac.jp (Y.N-I.) yiizumi@koto.kpu-m.ac.jp (Y.I.); mtkw@koto.kpu-m.ac.jp (M.W.); ysowa@koto.kpu-m.ac.jp (Y.S.); mimutoh@koto.kpu-m.ac.jp (M.M.)

[2] Department of Drug Discovery Medicine, Kyoto Prefectural University of Medicine, Kyoto 602-8566, Japan; m-hori@koto.kpu-m.ac.jp (M.H.); tsakai@koto.kpu-m.ac.jp (T.S.)

[3] Laboratory of Biopharmaceutics and Pharmacokinetics, Faculty of Pharmaceutical Sciences, Hiroshima International University, Hiroshima 737-0112, Japan; takakura@hirokoku-u.ac.jp (H.T.); oda@hirokoku-u.ac.jp (K.O.); n-mori@hirokoku-u.ac.jp (N.M.)

[4] Pathological and Biomolecule Analyses Laboratory, Faculty of Pharmacy, Kindai University, Osaka 577-8502, Japan; yamatetsu@phar.kindai.ac.jp (T.Y.)

* Correspondence: mimutoh@koto.kpu-m.ac.jp; Tel.: + 81 75 251 5539

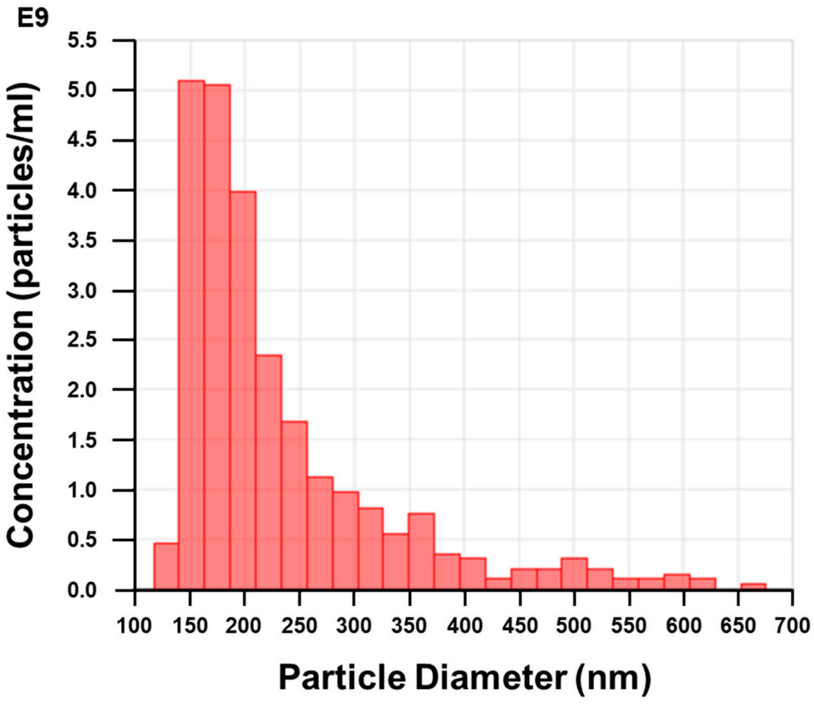

Figure S1. Analysis of the diameter and concentration for *Citrus limon L.* -derived nanovesicles by tunable resistive pulse sensing.

Table S1. *Citrus limon L.*-derived nanovesicle concentration by tunable resistive pulse sensing.

Particle concentration
2.50×10^{10} particles/ml

2.50×10^{10}: the total number of digits is 11, and the first three digits are "250".
